# Scaffolds for Osteochondral Lesions of the Talus: Systematic Review and Meta-Analysis of the Last Ten Years Literature

**DOI:** 10.3390/bioengineering11100970

**Published:** 2024-09-27

**Authors:** Francesca Veronesi, Simone Ottavio Zielli, Silvia Brogini, Elena Artioli, Alberto Arceri, Antonio Mazzotti, Cesare Faldini, Gianluca Giavaresi

**Affiliations:** 1 Surgical Sciences and Technologies, IRCCS Istituto Ortopedico Rizzoli, Via Di Barbiano 1/10, 40136 Bologna, Italy; francesca.veronesi@ior.it (F.V.); silvia.brogini@ior.it (S.B.); gianluca.giavaresi@ior.it (G.G.); 2 1st Orthopaedic and Traumatologic Clinic, IRCCS Istituto Ortopedico Rizzoli, Via Putti 1, 40136 Bologna, Italy; elena.artioli@ior.it (E.A.); alberto.arceri@ior.it (A.A.); antonio.mazzotti@ior.it (A.M.); cesare.faldini@ior.it (C.F.); 3 Department of Biomedical and Neuromotor Sciences (DIBINEM), Alma Mater Studiorum University of Bologna, 40126 Bologna, Italy

**Keywords:** systematic revision of the literature, talus, osteochondral regeneration, clinical studies

## Abstract

Scaffolds are widely used devices for the treatment of osteochondral lesions of the talus (OCLT), aimed at enhancing mechanical stability and fostering chondrogenic differentiation. A systematic review and meta-analysis were performed to evaluate the safety, and clinical and radiological results of scaffolds for OCLT management. On 2 January 2024, a search was performed in four databases (PubMed, Embase, Web of Science, and Scopus), according to PRISMA guidelines. The risk of bias in the included studies was also evaluated. Thirty clinical studies were included in the qualitative analysis: 12 retrospective case series, 3 retrospective comparative studies, 9 prospective case series, 1 prospective comparative study, and 1 Randomized Controlled Trial (RCT). Natural scaffolds, such as bilayer collagen (COLL)I/III and hyaluronic scaffolds, were the most employed. Only minor adverse events were observed, even if more serious complications were shown, especially after medial malleolar osteotomy. An overall clinical and radiological improvement was observed after a mean of 36.3 months of follow-up. Patient age and Body Mass Index (BMI), lesion size, and location were correlated with the clinical outcomes, while meta-analysis revealed significant improvement in clinical scores with hyaluronic scaffolds compared to microfracture alone. This study highlights the safety and positive clinical outcomes associated with the use of scaffolds for OCLT. In the few available comparative studies, scaffolds have also demonstrated superior clinical outcomes compared to microfractures alone. Nevertheless, the analysis has shown the limitations of the current literature, characterized by an overall low quality and scarcity of RCTs.

## 1. Introduction

Osteochondral lesion of the talus (OCLT) is defined as a lesion of the talar dome that comprises the joint surface hyaline cartilage and the subchondral bone [1] and was first described by Kappis in 1922 [2]. OCLTs frequently occur in young men patients (20–30 years) during sports activities [3], and the main causes are traumatic events, such as sprains or fractures [4]. Other causes can be attributed to degenerative joint disease, ischemia, ossification disorders, avascular necrosis, peripheral vascular disease, malalignment, hormonal, endocrine or genetic factors, alcohol abuse, or idiopathic causes [5,6]. OCLT is frequently observed in anterolateral or posteromedial locations of the talus [7] and is associated with variable symptoms such as chronic ankle pain, movement limitation, swelling, locking, and joint stiffness [8]. OCLT represents a challenge for orthopedic surgeons due to the intrinsic poor regenerative ability of the cartilage and the peculiar vascularization characteristics of the talus, leading to chronic ankle disability and early osteoarthritis if not properly treated [9].

Surgical treatments aim to restore the superficial hyaline cartilage, well integrated with the surrounding cartilage, and the subchondral bone to obtain proper support [10,11]. Over these decades, several different approaches have been developed, each with its own advantages and disadvantages; still, there is no consensus on which technique is superior to the others [4]. Based on lesion size, depth, location, and involvement of subchondral bone, treatments can be grouped into cartilage replacement therapy, such as Autologous Chondrocyte Implantation (ACI), allograft or autograft transplantation, and bone marrow stimulation techniques, such as microfracture (MF), and drilling [12,13,14]. Among these techniques, MF is one of the most used methods due to its ease of use and ability to deliver bone marrow mesenchymal stem cells (BMSCs) to the lesion site. Various studies have demonstrated that this technique tends to result in the formation of fibrous-type cartilage over time [15,16].

In 2008, Autologous Matrix-Induced Chondrogenesis (AMIC) was proposed: debridement, MF, and matrix (usually a collagen or hyaluronic acid membrane) are applied in a single step [17]. Other new techniques, developed recently, include Matrix-Induced Autologous Chondrocyte Implantation (MACI) [18], High-Density Autologous Chondrocyte Implantation (HD-ACI) [19], and Matrix-Associated Stem Cell Transplantation (MAST) [20].

The use of a scaffold is well-known to improve mechanical stability and promote tissue repair and regeneration by providing a stimulus for chondrogenic differentiation [21], thus promoting tissue repair and regeneration. Scaffolds are used to provide support, and a framework for the growth of new tissue and various matrices is being researched and utilized for the treatment of OCLT. They are divided into biosynthetic, biological, and hydrogels [22,23,24].

The choice of a scaffold depends on the specific characteristics of the lesion, the patient’s condition, and the surgeon’s preference. Biosynthetic scaffolds, such as polymers, are used as support for tissue regeneration. They are designed to mimic the natural structure of bone and cartilage and they can be absorbable or permanent, depending on the material used [22]. Biological materials derived from natural sources, such as collagen or extracellular matrix components, are used to provide a biological framework for tissue repair, promoting cell attachment, migration, and proliferation [23]. Hydrogels are three-dimensional networks of hydrophilic polymers that can retain a significant amount of water. They are utilized to transport cells or growth factors to the affected area and establish a suitable environment for tissue regeneration [24].

The aim of the present study was to systematically collect clinical studies that used scaffolds to treat OCLT over the past decade to determine the scaffolds’ safety and whether the incorporation of scaffolds enhances clinical results. Clinical and radiological results, as well as surgical complications, were analyzed. Additionally, the correlation between patient or lesion characteristics and clinical outcomes was highlighted. A meta-analysis was conducted to compare clinical outcomes between studies that combined scaffolds with MF and those that exclusively relied on MF.

## 2. Materials and Methods

### 2.1. Data Source and Searches

**Figure 1:**
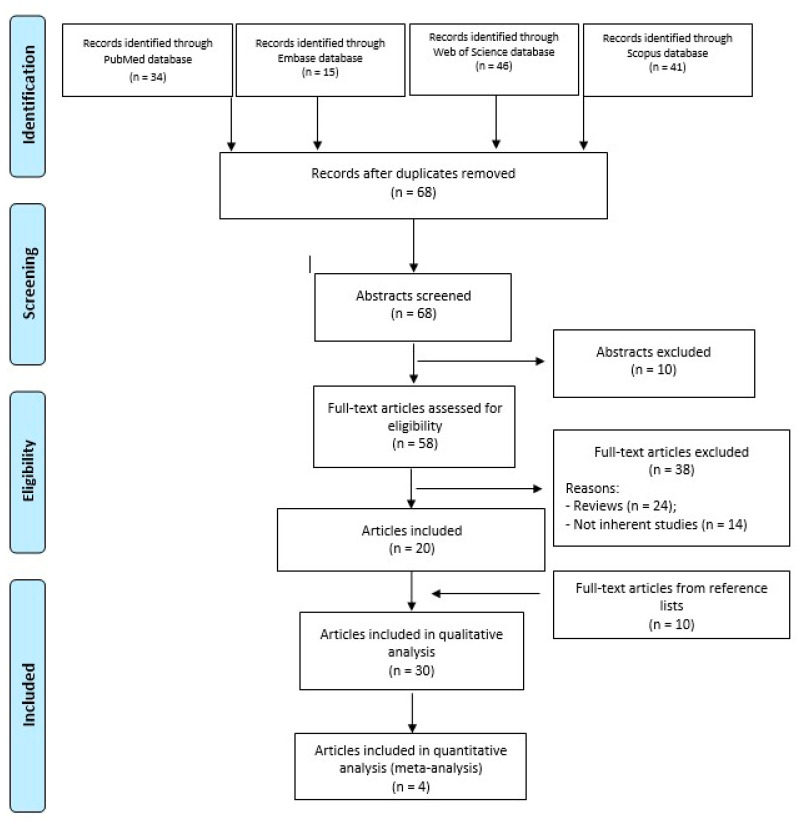
PRISMA flowchart of the study selection process.

A search was performed on 2 January 2024 in the following databases, with 10-year time limits, and without other filters: PubMed, Embase, Web of Science, and Scopus. The following string was applied for all the databases: (osteochondral defects) AND (scaffold) AND (ankle). Preferred Reporting Items for Systematic Reviews and Meta-Analyses (PRISMA) guidelines were used, and a flowchart of the study selection for data synthesis is reported in Figure 1.

### 2.2. Study Selection

Duplicates were removed through EndNote21. Then, the articles were screened by reading the title and abstract according to the following inclusion criteria: clinical studies of any level of evidence, written in English, on the surgical treatments that employed scaffolds for ankle osteochondral defects of all grades and sizes. Preclinical studies, reviews, book chapters, comments, or technical notes were excluded. Subsequently, the full texts of articles were read in case not enough information could be retrieved from the abstracts, using the inclusion and exclusion criteria listed above. The article selection process was independently performed by two authors (FV and SOZ) with disagreement on study eligibility solved by a third author (GG).

### 2.3. Data Extraction

Relevant data were independently extracted and collected using a standardized extraction form by two authors (FV and SOZ). The collected relevant data concerned reference, study type and blinding, study groups and scaffolds used, surgical approach, patients’ characteristics (number, age, sex, and body mass index—BMI), joint number, osteochondral defect size and grade, final follow-up (in months), and main results. Main complications and correlations between clinical results and patients/lesion characteristics were also collected.

### 2.4. Risk of Bias

The risk of bias in the studies was assessed independently by two authors (FV and SOZ) with disagreements resolved by consensus with a third author (GG), using the Dawns and Black checklist [25] and the modified Coleman methodology score [26] for all the included studies.

The Dawns and Black (minimum 0 to maximum 31) consists of 26 items distributed between five sub-scales: (1) Reporting (9 items); (2) External validity (3 items); (3) Bias (7 items); (4) Confounding (6 items); (5) Power (1 item) [25]. The modified Coleman methodology score takes into consideration 11 items in total: (1) Study size; (2) Mean follow-up; (3) Number of different surgical procedures included in each reported outcome; (4) Type of study; (5) Description of surgical procedure given; (6) Description of postoperative rehabilitation; (7) Inclusion MRI outcome; (8) Inclusion histological outcome; (9) Outcome criteria; (10) Procedure for assessing clinical outcomes; (11) Description of subject selection process [26].

### 2.5. Quantitative Synthesis and Statistical Analysis

The statistical analysis was conducted using the Jamovi project software (Version 2.3, 2022) obtained from https://www.jamovi.org (accessed on 29 July 2024). Eligibility for inclusion in the meta-analysis was contingent upon the availability of mean and standard deviation data for the collected parameters. In cases where mean and variance data were not directly provided but median, range, and sample sizes were available, we applied the method proposed by Hozo et al. [27] to derive the mean and variance values. The meta-analysis was performed when applicable to four or more studies. The analysis employed the standardized mean difference as the outcome measure, and a random-effects model was applied to the data.

## 3. Results

As shown in Figure 1, a total of 136 papers (34 in PubMed, 15 in Embase, 46 in Web of Science, and 41 in Scopus) were obtained through the 4 databases. After duplicate removal (n = 68), the other papers (n = 68) were evaluated by reviewing titles and abstracts according to the inclusion/exclusion criteria. Fifty-eight full-text articles were assessed for eligibility, and, among these, 38 papers were excluded because they were reviews (n = 24), and not inherent studies (n = 14), that regarded OCL of the knee, technical notes, in vivo or in vitro studies, or treatment without scaffolds application.

Twenty papers were included, and a further search was performed by reading the reference lists of these papers and a further 10 papers were considered. Thus, a total of 30 papers were included in the qualitative analysis and 4 of them in the quantitative synthesis (meta-analysis) (Table 1).

### 3.1. Study Type

**Figure 2:**
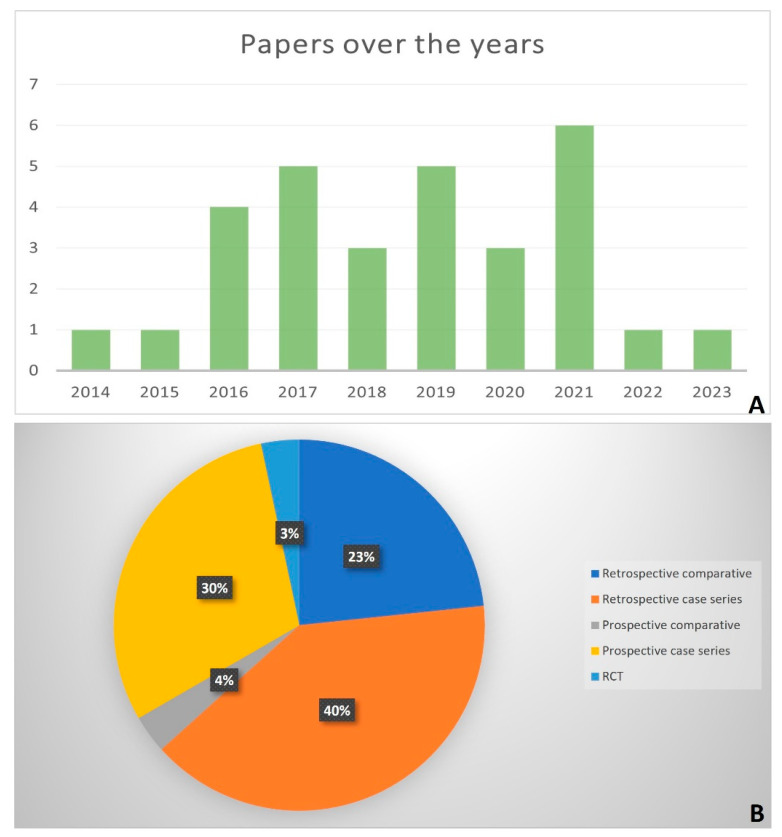
Histogram of the number of papers per year (2014–2023) (**A**); Pie chart of the percentages of the type of studies (**B**).

The years of publication of the included studies went from 2014 to 2023, most of them were published in 2021 (n = 6/30) (Figure 2A). Nineteen studies were retrospective (n = 12 case series, n = 7 comparative) [28,29,30,31,32,33,34,35,36,37,38,39,40,41,42,43,44,45,46], and the other 11 studies were prospective (n = 9 case series, n = 1 comparative, and n = 1 randomized controlled trial-RCT) [17,19,47,48,49,50,51,52,53,54,55] (Figure 2B). Most of them (n = 22) were not blinded [17,28,29,30,31,32,33,35,36,37,38,39,42,44,46,48,49,50,51,52,53,54], and the others were single-blinded (usually with respect to a radiologist) (n = 7) [19,34,41,43,45,47,55], while in one study, the blinding was not reported [40] (Table 1).

### 3.2. Risk of Bias

The risk of bias, evaluated with the Downs and Black checklist showed an overall poor quality of the included studies, with an average score of 18.6 (range: 17–23) (Table 2). In addition, the average modified Coleman methodology score was 49 (range: 31–60), showing that the methodological quality of the studies was quite modest (Table 2).

### 3.3. Patients and Lesion Characteristics

There were 842 patients treated with scaffolds, 114 treated with only surgery without treatment, and finally, 38 patients treated with platelet-rich-plasma (PRP) injection after surgery. In eight out of thirty studies, OCLTs were bilateral [32,37,42,43,48,52,53,55]. The ratio between males and females was 1.3 and the mean age of the patients ranged from a minimum of 23 years to a maximum of 52 years and only one study did not report age of the patients [40]. Eighteen out of thirty studies reported BMI values, and they ranged from a minimum mean of 24.6 kg/m^2^ to a maximum mean of 33.6 kg/m^2^ (Table 1).

The OCL grade was not reported in some studies (n = 14) [28,30,31,32,38,43,45,46,47,48,51,53,54,55], while in the other studies, Hepple classification was the most employed one (n = 5) [36,37,41,49,52], followed by Berndt, Harty, and Loomer (n = 4) [17,29,42,50], ICRS (n = 2) [19,29], modified classification based on MRI (n = 2) [33,39], Giannini (n = 1) [34,35], Outerbridge (n = 1) [40], Mintz (n = 1) [42], and Bristol (n = 1) [44] classifications. The Online Resource tables (S1–S7) report these classifications (they go from 0/1 to 4/5), with the higher value corresponding to a worse appearance of the lesion [56,57,58,59].

Also, the OCL area varied greatly between studies from a minimum of 0.9 cm^2^ [28,36,43,46,53] to a maximum of 4 cm^2^ [40], while some authors did not report dimensions [37,49,55] (Table 1).

### 3.4. Surgical Approach

Regarding surgical approaches, 14 authors employed arthroscopy (AR) [17,32,34,36,39,40,41,42,43,44,45,46,53,55], while 8 authors applied a medial malleolar osteotomy (MMO) [28,29,35,37,38,47,50,51], and 2 authors utilized an open technique [48,49]. In addition, four authors used a combination of AR for some patients and MMO for others within the same study [19,30,52,54]; in one study, AR was adopted for some patients and open surgery for others [31], and finally, in one study, surgeons performed open surgery for some patients and MMO for others [33] (Table 1).

### 3.5. Qualitative Analysis

#### 3.5.1. Retrospective Case Series

Twelve studies were retrospective case series [28,29,30,31,32,33,34,35,36,37,38,39], the first eight studies used natural or synthetic scaffolds, and the other four the BG (Table 1). The clinical results were evaluated through the American Orthopedic Foot and Ankle Society (AOFAS), Visual Analogue Scale (VAS), Tegner, Foot and Ankle Ability Measure (FAAM), and Foot and Ankle Outcome Score (FAOS) scores. AOFAS score (0–100 points) takes into consideration pain (40 points), function (50 points), and alignment (10 points), and higher values indicate a better state of the ankle [60]. VAS measures pain intensity (0 = no pain; 10 = pain as bad as it could possibly be) [61]. The Tegner activity scale is a one-item score that grades activity based on work and sports activities on a scale of 0 to 10. Zero represents disability because of knee problems and ten represents national or international level soccer [62]. FAAM is a self-report outcome instrument and a 29-item questionnaire divided into two subscales: the 21-item Activities of Daily Living Subscale and the Foot and Ankle Ability Measure (0–84) and the 8-item Sports Subscale (0–32). Higher scores represent higher levels of function for each subscale, with 100% representing no dysfunction [63]. FAOS (0–100) is a patient-reported outcome measure designed to assess the functional status and quality of life. It includes five subscales that evaluate different aspects (pain, other symptoms, function in daily living, function in sports and recreation, and foot- and ankle-related quality of life) and higher scores indicate better outcomes [64].

In three studies, a collagen I/III (COLL I/III) bilayer matrix was used alone [30] or combined with an autologous bone graft (BG) [28], or matrix-augmented bone marrow stimulation (M-BMS) [29]. After a mean of 12, 24, and 56 months, a significant decrease in VAS [28,30] and a significant increase in AOFAS [28,30], Tegner [28], FAAM Activity Daily Live (FAAM-ADL), FAOS-Pain, stiffness, ADL, Sport and Quality of Life (QoL) [29], and a 79% return to sport [28] were observed. Magnetic Resonance Observation of Cartilage Repair Tissue (MOCART) values, observed in two studies, were 61 ± 21 [28] and 54 ± 14 [29] at the end of the follow-up (Table 1). This 9-part and 29-item scoring system yields a final cartilage repair tissue score ranging from 0 to 100 points, where 0 points indicate the worst imaginable score, and 100 points represent the best imaginable score. A score below 27 is generally associated with poorer outcomes and reduced quality of cartilage repair tissue [65].

Five further studies used scaffolds based on hyaluronic acid (HA) [31], hyaluronan cultured with chondrocytes [32], polylactide-co-glycolide, calcium sulfate, and polyglycolide fibers [33], and polyglycolic acid-hyaluronan (PGA-HA) [34]. All studies observed significantly higher AOFAS results and lower VAS values after a mean of 20.3 [31], 87.2 [32], 90 [33], and 33.8 [34] months, respectively. Also, MaioRegen (FinCeramica Faenza, Faenza, Italy) reached the same results after a mean of 30 months [35].

MOCART values had a mean of 61.1 [33] and 64.2 [34] and showed an increase during time [35] (Table 1).

In four studies, autologous or allogenic BG was employed: autologous osteochondral calcaneus BG in one study [36], autologous graft harvested from the ipsilateral talar articular facet in another study [37], autologous cancellous bone from the distal tibial metaphysis in a third study [38], and a commercially available and Food and Drug Administration-approved particulated juvenile cartilage allograft transplantation (PJCAT) in a fourth study [39]. In all studies, significant decreases in VAS and increases in AOFAS scores were noted after an average follow-up period of 18.9 [36], 66 [37], 25.3 [38], and 41.8 [39] months. Additionally, significant improvements were observed also in Tegner [36]. Only one study evaluated MOCART, with a value of 68 ± 14.8 at the end of follow-up [36] (Table 1).

#### 3.5.2. Retrospective Comparative Studies

Three retrospective studies were comparative [40,43,46] (Table 1). They employed AOFAS and VAS to evaluate clinical results.

One study compared the HA-based scaffold with the Chitosan-based one [40], the second COLL I/III scaffold and autologous BG with autologous BG alone [43], and the third PJCAT, added with juvenile allogenic chondrocyte implantation with autologous bone marrow aspirate (JACI-BMAC), with BMAC alone [46].

In the first and second studies, the AOFAS score significantly increased and VAS significantly decreased in both groups without differences between the two groups [40,43]. In the third study, FAOS, VAS, and lesion regeneration significantly improved in the presence of both treatments, with a reparative tissue that exhibited a fibrocartilage composition [46].

Better MOCART results were observed in patients treated with autologous AB, in comparison to those treated with scaffold and autologous BG [43], while in the other two studies, both treatments showed the same MOCART score [40,46] (Table 1).

#### 3.5.3. Prospective Case Series

Nine studies were prospective case series [17,19,47,48,49,50,51,52,53] (Table 1). The clinical evaluations were performed with AOFAS, VAS, FAOS, Ankle Osteoarthritis Scale (AOS), and foot function index (FFI). The AOS score is a reliable and valid self-assessment instrument that specifically measures patient symptoms (9 items) and disabilities (9 items) related to ankle arthritis, with a total of 18 items. Higher scores indicate worse pain and disability [66]. FFI is a self-administered index consisting of 23 items divided into 3 sub-scales (pain 0/90, disability 0/90, and activity restriction 0/50), with higher scores indicating worse pain [67].

In six out of nine studies, COLL I/III matrix was added with autologous BG [17,48,49,50,51] or with high-density chondrocytes [19] and, after a mean of 24 [17,19], 33.5 [49], 39.5 [50], 46.4 [51], and 60 [48] months, VAS [17,19,49,50,51] significantly decreased, while AOFAS and FFI significantly increased [17,19,49,50,51].

MOCART, evaluated in five studies, increased during time [17,49] and the values were 52.7 ± 15.9, 69.5 ± 16.7, and 72.3 ± 16.2 [19,50,51] (Table 1).

In other two studies, an aragonite-based bi-phasic scaffold [52,53], and a matrix of tricalcium phosphate (βTCP) filled with human recombinant PDGF (rhPDGF) [53] significantly increased FAOS [52] and AOS [53] and significantly reduced VAS [53] after 26 [52] and 6 [53] months.

MOCART showed a mean of 67.8 in one study [53] (Table 1).

Finally, only one study showed no complete regeneration of the subchondral bone and no improvement in the AOFAS score and MOCART after a mean of 30 months from the MaioRegen application [47] (Table 1).

#### 3.5.4. Prospective Comparative Studies

Two studies were prospective comparative [54,55], one of which was RCT [55] (Table 1). The clinical evaluations were performed with AOFAS, VAS, and FAAM.

One study employed autologous bone chips covered with COLL I/III or HA-based scaffolds and compared the two different surgical approaches (MMO vs. AR) [54]. After a mean of 22 months, both techniques significantly increased AOFAS and reduced VAS, without differences between them. Also, MOCART did not report differences between the groups of treatment [54] (Table 1).

The second study compared patients treated with or without PRP injections. After a mean of 16.2 months, better AOFAS, FAAM, and VAS values were observed in patients treated with PRP [55] (Table 1).

### 3.6. Safety and Complications

The main complications were taken into consideration in some studies in which AR [17,32,34,36,39,40,41,42,43,45,46,53], MMO [28,35,37,38,50,51], or open surgery [49] were performed with or without scaffolds (Table 3). The complications were related to the surgical intervention and not to the use of the scaffold itself.

AR, alone without scaffolds, induced ankle hematoma (6.5%) [41], transient neuropraxia in the dorsal branch of the superficial peroneal nerve (4.5%), and pain (9%) [42].

The use of scaffolds associated with AR induced ankle swelling (9.4% or 3.1%) [34,41], ankle hematoma (6.3%) [41], superficial skin infection of the arthroscopic portal (4.3% or 2.1%) [43], and synovial fistula (2.1%) [43]. The re-operation rates went from 2.4% [40] to 30% [46]. Four studies evidenced the absence of complications in the presence of osteochondral autograft [36], chitosan-based scaffold [42], polyglycolic acid-hyaluronan scaffold [45], and β-TCP matrix + rhPDGF [53].

As regards the MMO technique, which consistently involved the use of a scaffold, observed complications included delayed union (3%) [28], superficial wound infection (6.5%), numbness at the distribution area of superficial peroneal nerve (2.2%), occasional ache over the anteromedial aspect of ankle (4.3%) [37], and iatrogenic lesion of the posterior tibial tendon (10%) [51]. Re-operation was found in one study in 25% of patients [35]. Two studies found no complications after the application of autologous BG [38] and COLL I/III + autologous BG [50].

Finally, open surgery with scaffold induced transient post-operative irritation of the deep peroneal nerve (4.3%), painful arthrofibrosis (4.3%), and persistent pain (4.3%) [49].

### 3.7. Correlations

Table 4 shows studies correlating age, BMI, gender, symptom duration, p.o. pain, lesion size, and location with clinical scores.

Among the 30 studies, 8 performed these correlations [28,29,30,32,38,48,50,51]:

Age < 40 years was positively correlated with high AOFAS score at different follow-ups (*p* = 0.046 at 12 months; *p* = 0.05 at 36 months; *p* = 0.008 at final follow-up) [32] and age ≥ 45 years with lower pain (*p* = 0.048) [50]. In three studies, age was not correlated with clinical scores [28,29,30].

BMI > 30 was positively correlated with lower AOFAS scores (*p* = 0.003) and higher VAS (*p* = 0.031) [50], while two studies did not find correlations between BMI and clinical scores [28,29].

High p.o. pain was positively correlated with low AOFAS (*p* = 0.004) [50].

Lesion size was positively correlated with FFI pain (*p* = 0.012) and FFI function (*p* = 0.016) [48], and lesion size ≥ 3 cm^3^ with low AOFAS (*p* = 0.041) [50]. In four studies, no correlations were found between lesion size and clinical scores [28,30,38,51]. Also, the lateral lesion was positively correlated with high AOFAS at different follow-ups (*p* = 0.007 at 12 months, *p* = 0.001 at 36 months) [32], while two studies did not find correlations [29,30].

Gender and symptom duration did not appear to influence clinical results [29].

### 3.8. Quantitative Analysis: MF Alone vs. MF and Scaffold

**Figure 3:**
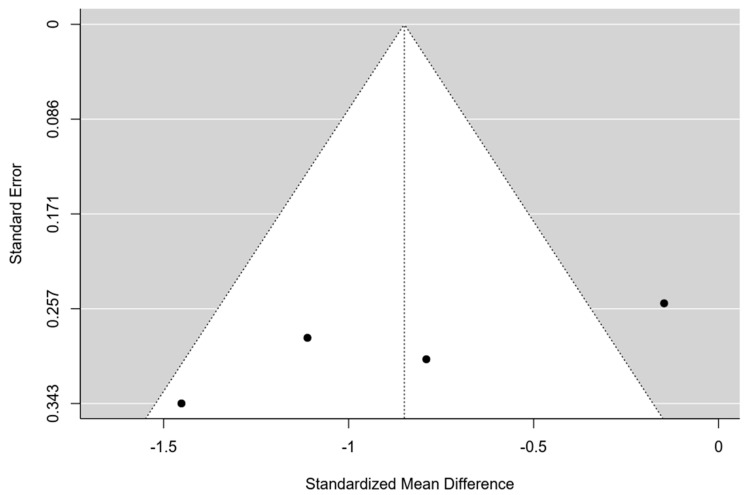
Funnel plot of the 4 studies included in the Meta-analysis.

**Figure 4:**
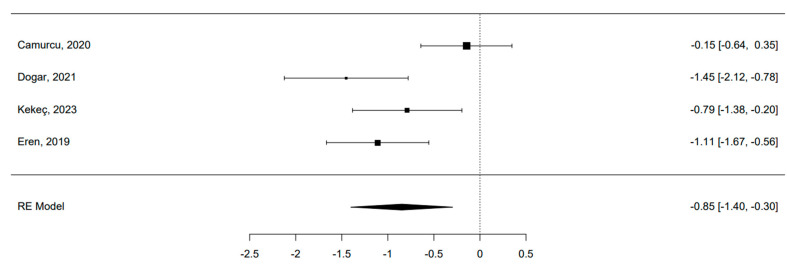
Forest plot of the 4 studies included in the Meta-analysis [41,42,44,45]. The pooled effect size indicates a statistical significative superiority of scaffolds compared to MF.

Only four studies were eligible for meta-analysis [41,42,44,45]. The 95% prediction interval for true outcomes ranged from −1.94 to 0.24, suggesting potential variability in study-specific effects. One study [41] stood out as a potential outlier, with a studentized residual exceeding ±2.50. A funnel plot is shown in Figure 3. The estimated average standardized mean difference was −0.85 (95% CI [−1.40, −0.30]), signifying a noteworthy enhancement in the clinical score among patients utilizing a hyaluronic scaffold compared to the control group treated solely with microfractures (z = −3.01, *p* = 0.002) (Figure 4). The meta-analysis demonstrated a substantial average improvement in the AOFAS clinical score for patients employing a hyaluronic scaffold at the site of the osteochondral lesion in comparison to the control group treated with microfractures. Nonetheless, significant heterogeneity and potential variability in individual study effects were observed.

## 4. Discussion

This systematic review and meta-analysis collected clinical evidence on the safety and the overall positive results of scaffolds for the management of OCLTs.

The osteochondral (OC) unit is composed of both cartilage and subchondral bone, which, although histologically different, are biologically and functionally linked, one influencing the other both in physiological and pathophysiological processes. An effective regenerative solution should properly regenerate bone, cartilage, and the bone–cartilage tidemark [68]. So, the preclinical and clinical challenge is to obtain an optimal scaffold reflecting the complexity and hierarchical, topographical, and mechanical features of the OC tissue.

Two scores were used to assess the risk of bias in the included studies. Both scores highlighted the poor quality of the included studies, with only one RCT and few comparative studies, so it is difficult to make a comparison between different treatments or different surgical techniques. More precisely, all the studies (100%) were unable to blind the subjects to the intervention they had received, and the subjects were not randomized to intervention groups. In addition, 90% of the studies did not describe the characteristics of patients lost to follow-up; in 97% of the studies, the subjects who asked to participate in the study were not representative of the entire population from which they were recruited, 77% of the studies were not able to blind those measuring the main outcomes of the intervention, 73% of the studies had no adequate adjustment for confounding in the analyses from which the main findings were drawn, 93% of the studies did not take into account the losses of patients to follow-up, and 77% of the studies had no sufficient power to detect a clinically important effect where the probability value for a difference being due to chance is less than 5%. Modified Coleman methodology score showed that in 67% of the studies, the number of lesions was under 40, 63% of the studies were retrospective cohort ones, and 87% of the studies did not perform histology.

All types of scaffolds, natural or synthetic ones, improved all the clinical and radiological outcomes in all types of OCLTs from the peri-implantation period to the final follow-up. Among them, the most employed was the bilayer COLL I/III (9/21 studies) scaffold [17,19,28,29,30,48,49,50,51], alone [30] or combined with autologous BG [17,28,48,49,50], BMC [29,51], or chondrocytes [19]. COLL is a natural component of skeletal tissues and is able to induce cartilage repair, and graft stabilization, so reduces graft failure [69]. This matrix, secured in the lesion site through fibrin glue, has always been associated with the AMIC procedure since 1999 to stabilize the super clot on top of the lesion after MF, avoiding the loss of MSCs into the joint space and, at the same time, assuring the chondrogenic differentiation of these cells [70,71]. Other natural matrices used by some authors for AMIC were HA-based or hyaluronan ones [31,32].

Osteochondral autograft, autologous BG, or PJCAT allograft were also largely employed [36,37,38,39]. Among them, PJCAT has recently gained acceptance as a treatment option for OCLTs, since the young age of the donors shows immunologic privilege, with enhanced potential to proliferate and fill a recipient site cartilage defect better than a mature tissue, with a hyaline-like cartilage [72,73]. This type of scaffold is employed especially in large OCLTs and in the presence of subchondral cysts [71]. However, as observed in the present review, they were used in lesions with similar size and grades to those in which the COLL I/III matrix was applied.

Only one scaffold did not produce the desired results: MaioRegen showed no subchondral bone regeneration, no clinical and radiological improvements [47], and high rates of treatment failure [35].

The HA-based scaffold was compared to the chitosan-based one [40], COLL I/III matrix and autologous BG to matrix alone [43], and PJCAT and BMAC to BMAC alone [46]. We have already talked about the bilayer COLL I/II and the PJCAT above. Chitosan derived from the exoskeleton of crustaceans contains large amounts of glucosamine polysaccharide and is characterized by low toxicity, high biocompatibility, biodegradability, and adhesion to tissues, promoting hyaline cartilage regeneration [74]. The two types of scaffolds demonstrated the same clinical and radiological results at the final follow-up.

Regarding surgical techniques employed in the included studies, three approaches were used: AR was the most employed [17,19,30,31,32,34,36,39,40,41,42,43,44,45,46,52,53,54,55], followed by open surgery [31,33,48,49] and MMO [19,28,29,30,33,35,37,38,47,50,51,52,54]. MMO and AR showed no differences at the final follow-up [54].

The choice between an arthroscopic MF, open surgery, or MMO depends on several factors, including the localization, the specific nature of the OCLT, as well as the surgeon’s preferences. Arthroscopic MF is a minimally invasive procedure; however, it is not exempt from potential complications, such as the risk of neurovascular injuries and increased surgical complexity [75]. Handling of the scaffold may pose additional challenges, as does ensuring its correct positioning.

On the other hand, an open approach provides a better understanding of the lesion and greater ease in placing scaffolds. However, it comes with longer recovery times compared to arthroscopy, and on paper, it carries a higher risk of surgical wound complications [76]. MMO is a surgical procedure that involves cutting and repositioning the medial ankle malleolus to access the postero-medial OCL that would otherwise be challenging to reach. This type of access is undoubtedly the most invasive and is burdened by potential complications, such as delayed healing, pseudoarthrosis, or the development of secondary arthritis in cases of malunion. Despite the possibility of significantly reducing these complications with the use of correct surgical techniques, this review has highlighted that the risk of delayed consolidation is not entirely eliminated. Additionally, one author reported an iatrogenic injury to the posterior tibial tendon secondary to MMO, a potentially dangerous complication with a negative prognosis if not correctly identified and addressed.

It is particularly interesting to analyze complications, even though they have not been systematically reported by all the authors [17,28,32,34,35,36,37,38,39,40,41,42,43,45,46,49,50,51,53]. They were very variable among the studies, and overall minor adverse events, such as superficial wound infection or numbness, usually at the distribution area of the superficial or deep peroneal nerve, were the most reported ones. However, as mentioned earlier, more serious complications, such as an iatrogenic tendon injury, have also been reported, mostly related to MMO. It is important to note that the most serious adverse events were due to the surgical technique and not to the use of the scaffold itself. The highest re-operation rate was observed in the presence of PJCAT and JACI-BMAC or BMAC alone, after the AR technique [46], and COLL I/III and MaioRegen, after MMO [28,35].

The correlations between lesion/patients’ characteristics and clinical results were also evaluated [28,29,30,32,38,48,50,51]: age, BMI, gender, symptom duration, p.o. pain, lesion size, and location were correlated with FFI, pain, and AOFAS. The age of the patients is very young (36.4 ± 5.3 years) and there is an almost homogeneous distribution between males and females, according to the literature [3]. It was observed that age under 40 years was correlated with high AOFAS and high pain, BMI > 30 with low AOFAS and high VAS, high p.o. pain with low AOFAS, lesion size ≥ 3 cm^3^ with low AOFAS and FFI, and lateral lesion with high AOFAS [32,48,50]. On the other hand, gender and symptom duration seemed not to be correlated with clinical results [29].

A meta-analysis was conducted with four comparative studies [41,42,44,45]. A comparison between AR alone and AR with scaffolds was performed. It revealed a substantial average improvement in the AOFAS clinical score for patients using a hyaluronic scaffold at the osteochondral lesion site compared to the control group treated with MF. While a previous systematic review has sought to address if scaffold-based therapy is effective for OCLT [77], our study marks the first attempt to conduct a meta-analysis on clinical outcomes in studies comparing scaffolds to sole AR, probably considered the standard treatment for these types of lesions. This preference arises from the compromise between the complexity of the surgical procedure, cost considerations, and desired clinical outcomes. Despite the inherent limitations associated with retrospective comparative study designs, the obtained results are promising. However, a noteworthy limitation persists: comparative studies constituted a minority within the overall pool of analyzed studies. Furthermore, considerable heterogeneity and potential variability in individual study effects were observed. Additionally, the lack of sufficient radiological data hindered a comprehensive analysis that could have incorporated this crucial variable.

Several limitations were identified in the included studies. These include the overall low methodological quality, particularly due to the absence of power analyses, blinding, and randomization. Furthermore, the lack of RCTs and the limited number of comparative studies significantly weaken the strength of the available evidence. The heterogeneity in the size and grade of OCLT across the studies, as well as inconsistencies in how lesion dimensions are reported, further complicate meaningful comparisons and limit the generalizability of the findings. As a result, no definitive conclusions can be drawn regarding the optimal scaffold for treating OCLTs of varying stages and dimensions.

Moreover, only four studies met the criteria for inclusion in the meta-analysis, reflecting a relatively small sample size. These studies predominantly employed a retrospective design, and the presence of an outlier in the funnel plot suggests potential issues related to study distribution and heterogeneity. Nevertheless, this meta-analysis represents one of the few investigations into OCLTs of the talus and, to the best of our knowledge, the first to focus specifically on the application of scaffolds for this type of injury.

However, several critical issues remain unresolved in the current research. One major challenge is the composition of the scaffolds themselves, which still likely fails to replicate the complex architecture and function of the osteochondral unit. This issue is closely related to the difficulty in recreating an appropriate subchondral bone microenvironment that facilitates the correct differentiation of precursor cells within the lesion site [78]. The current materials may not sufficiently mimic the biomechanical or biological environment necessary for successful repair.

Additionally, the paucity of studies evaluating outcomes from a radiological perspective limits our understanding of the long-term efficacy of scaffold-based treatments. Only a few studies have utilized comprehensive imaging modalities, such as the MOCART score, to assess the quality of repair tissue. Radiological data are essential for correlating clinical improvements with actual tissue regeneration, and future research should place greater emphasis on these objective measures.

Finally, significant technical challenges persist regarding the application of scaffolds in arthroscopic procedures, particularly for areas of the talus challenging to reach, such as the postero-medial one. The complexity of arthroscopic maneuvers and the limited accessibility of certain OCLs pose substantial barriers to the widespread adoption of scaffolds in clinical practice. Therefore, any future scaffold designs must prioritize ease of handling and adaptability to arthroscopic techniques, ensuring their successful use in minimally invasive surgeries.

## 5. Conclusions

This systematic review and meta-analysis provide insights into the safety and overall positive outcomes of scaffold use in OCLT surgery. Due to the paucity of comparative studies and the lack of RCT studies, it is difficult to draw a conclusion on the best scaffold employed for OCLT. Several different scaffolds have been employed, and among them, the natural ones, including bilayer COLL I/III and hyaluronic scaffolds and autologous BG were the most employed. The reported complications rarely correlated with the scaffold but rather with the surgical procedure, and generally included superficial skin infections and irritation to the peroneal nerves. Although very rare, it is important to note that more serious complications were observed, especially in relation to MMO, and not to the scaffold employed. Age, BMI, lesion size, and location correlated with clinical outcomes, emphasizing the importance of patient and lesion characteristics in treatment success. Finally, the meta-analysis highlighted a substantial enhancement in the AOFAS clinical score with hyaluronic scaffolds. However, the literature’s limitations, marked by low-quality studies and a scarcity of RCTs, underscore the need for more robust research.

## Figures and Tables

**Table 1:** Characteristics of the clinical studies on scaffold used for the treatment of OCLT. The studies are divided into (1) retrospective case series; (2) retrospective comparative studies; (3) prospective case series; (4) prospective comparative studies. Inside each group, the studies were divided according to scaffold type.

Article	Study Type Blinding	Groups and Scaffolds	Surgical Approach	Pts/Ankle	Age (Mean + SD)	Sex (M/F)	BMI (Mean + SD)	OCL Surface or Volume (cm^2^ or cm^3^); Grade	Final f-up (m)	Main Results
Weigelt, 2019 [28]	Retrospective case series NO	Group (1): COLL I/III + autologous BG	MMO	33/33	Mean 35.1	19/14	26.8 ± 4.3	0.9 ± 0.5 cm^2^; n.r.	Mean 56.4	Group (1): ↓ VAS; ↑ AOFAS, Tegner; 79% returned to their previous sports levels. MOCART = 61 ± 21
Gottschalk, 2021 [29]	Retrospective case series NO	Group (1): COLL I/III + M-BMS	MMO	45/45	34 ± 24	22/23	Mean 26 kg/m2	1.7 ± 1.3 cm^2^, 1 ± 1.38 cm^3^; II–IV grade (ICRS classification); II–V grade (Berndt, Harty, and Loomer classification)	12	Group (1): ↑ FAAM-ADL, FAOS-Pain, FAOS-Stiffness, FAOS-ADL, FAOS-Sport, and FAOS-QoL. MOCART = 54 ± 14
Ayyaswamy, 2021 [30]	Retrospective case series NO	Group (1): COLL I/III	AR or MMO	25/25	Mean 36	14/11	n.r.	Mean 1.75 cm^2^; n.r.	Mean 24	Group (1): ↑ AOFAS; ↓ VAS
Yontar, 2019 [31]	Retrospective case series NO	Group (1): HA-based	AR or Open	20/20	Mean 32.9	14/6	n.r.	1 ± 0.5 cm^2^; n.r.	Mean 20.3	Group (1): ↑ AOFAS; ↓ VAS
Giannini, 2014 [32]	Retrospective case series NO	Group (1): Hyaluronan + Chondrocytes	AR	46/50	31.4 ± 7.6	29/17	n.r.	Mean 1.6 cm^2^; n.r.	87.2 ± 14.5	Group (1): ↑ AOFAS
Di Cave, 2017 [33]	Retrospective case series NO	Group (1): Polylactide-co-glycolide, calcium sulphate, polyglycolide fibers	Open or MMO	12/12	Mean 38.6	9/3	n.r.	n.r.; 3–5 stages (modified classification based on MRI)	Mean 90	Group (1): ↑ AOFAS; ↓ VAS. MOCART average 61
Kanatlı, 2017 [34]	Retrospective case Series Single-blind (radiologist assessors)	Group (1): PGA-HA	AR	32/32	38 ± 12	21/11	28.6 ± 4.3	2.5 ± 0.8 cm^2^; II–IV types (Giannini classification)	33.8 ± 14.0	Group (1): ↑ AOFAS. MOCART = 64 ± 12
Albano, 2017 [35]	Retrospective case series NO	Group (1): MaioRegen	MMO	16/16	42.6 ± 18.4	8/8	26.3 ± 5.2	>1.5 cm^2^; II or IIA type (Giannini classification)	30 ± 16.9	Group (1): ↓VAS; ↑ AOFAS, MOCART
Li, 2021 [36]	Retrospective case series NO	Group (1): Osteochondral autograft	AR	24/24	39.8 ± 12.9	15/9	25.6 ± 2.4	n.r.; Stage V (Hepple classification)	18.9 ± 11.8	Group (1): ↑ AOFAS, Tegner, KAFS; ↓ VAS. MOCART = 68 ± 15
Georgiannos, 2016 [37]	Retrospective case series NO	Group (1): Autologous BG	MMO	46/48	36.2 ± 8.1	37/9	n.r.	n.r.; Stage III–V (Hepple classification)	Mean 66	Group (1): ↑ AOFAS; ↓ VAS
Sawa, 2018 [38]	Retrospective case series NO	Group (1): Autologous BG	MMO	12/12	Mean 35.9	7/5	n.r.	n.r.; n.r.	Mean 25.3	Group (1): ↑ AOFAS
Heida, 2020 [39]	Retrospective case series NO	Group (1): PJCAT allograft	AR	33/33	32.3 ± 6.8	26/7	28.3 ± 3.8	1.3 ± 0.5 cm^2^; 3–5 stage (modified classification based on MRI)	41.8 ± 19.4	Group (1): ↑AOFAS; ↓ VAS
Akmeşe, 2020 [40]	Retrospective comparative n.r.	Group (1): HA; Group (2): Chitosan	AR	81/81	n.r.	36/45	n.r.	1–4 cm^2^; III or IV grade (Outerbridge classification)	24	Groups (1), (2): ↑ AOFAS; ↓ VAS. Group (1): =AOFAS, VAS, MOCART than group (2)
Camurcu, 2020 [41]	Retrospective comparative Single-blind (radiologist assessor)	Group (1): Chitosan-glycerol phosphate/blood; Group (2): no treatment	AR	63/63	Mean 40.3	29/34	Mean 30.1	Group (1): 1.6 ± 0.2 cm^2^; Group (2): 1.5 ± 0.2 cm^2^; Stage II–IV (Hepple classification)	32 ± 13	Groups (1), (2): ↑ AOFAS; ↓ VAS. Group (1): =AOFAS, MOCART than Group (2); ↑ VAS function than Group (2)
Dogar, 2021 [42]	Retrospective comparative NO	Group (1): Chitosan; Group (2): PRP; Group (3): no treatment	AR	62/76	37.72 ± 13.31	31/31	Mean 26.30	Group (3): Mean 1.5 cm^2^; Group (2): Mean 1.48 cm^2^; Group (1): Mean 1.92 cm^2^; 2–5 stages (Modified Berndt Harty radiologic classification, Mintz classification)	26.2 ± 18.4	Groups (1), (2):↑ AOFAS. Group (3): ↓ VAS; ↑ FAAM overall pain, 15-min walking, and running function. Group (1): ↓VAS; ↑AOFAS, FAAM than groups (2), (3)
Gorgun, 2022 [43]	Retrospective comparative Single-blind (radiologist assessor)	Group (1): COLL I/III + autologous BG; Group (2): Autologous BG	AR	94/188	Mean 32	49/45	n.r.	Mean 1 cm^3^; n.r.	69.3 ± 20.7	Groups (1), (2): ↑AOFAS; ↓VAS. Group (1): =AOFAS and VAS. Group (1): ↓ MOCART than group (2)
Kekeç, 2023 [44]	Retrospective comparative NO	Group (1): PGA-HA-based CFS; Group (2): no treatment	AR	47/47	22.8 ± 2.3	29/18	23.7 ± 4.8	2.1 ± 0.3 cm^2^; 4–5 stage (Bristol)	36.2 ± 5.6	Groups (1), (2): ↑ AOFAS. Group (1): ↑ AOFAS, MOCART than Group (2)
Eren, 2019 [45]	Retrospective comparative Single-blind (Clinical assessors)	Group (1): Bio-absorbable polyglycolic acid-hyaluronan; Group (2): no treatment	AR	62/62	41 ± 13	35/27	Mean 27.4	Group (2): Mean 1.65 cm^2^, Group (1): Mean 1.97 cm^2^; n.r.	36.1 ± 14.9	Groups (1), (2): ↑ AOFAS. Group (1): ↑ AOFAS than group (2)
Karnovsky, 2018 [46]	Retrospective comparative NO	Group (1): PJCAT + JACI-BMAC; Group (2): BMAC	AR	50/50	Mean 37.2	23/27	n.r.	Group (2): Mean 0.8 cm^2^, Group (1): Mean 1.2 cm^2^; n.r.	Mean 30.9	Groups (1), (2): ↑ FAOS; ↓ VAS; =MOCART; fibrocartilage reparative tissue.
Christensen, 2016 [47]	Prospective case series Single-blind (radiologist assessor)	Group (1): MaioRegen	MMO	8/8	27 ± 7	5/3	n.r.	3.0 ± 1.9 cm^2^; n.r.	Mean 30	Group (1): No complete regeneration of the subchondral bone. No improvement in the MOCART and AOFAS
Gottschalk, 2017 [48]	Prospective case series NO	Group (1): COLL I/III + autologous BG	Open	21/37	37 ± 15	13/8	26 ± 5	1.4 ± 0.9 cm^2^; n.r.	60	Group (1): ↓ FFI
Galla, 2019 [49]	Prospective case series NO	Group (1): COLL I/III + autologous BG	Open	23/23	35.6 ± 13.9	15/8	n.r.	n.r.; Stage II, III, V (Hepple’s classification)	33.5 ± 10.4	Group (1): ↓VAS; ↑FFI; = MOCART during time
Kubosch, 2016 [50]	Prospective case series NO	Group (1): COLL I/III + autologous BG	MMO	17/17	38.8 ± 15.7	9/8	Mean 27.44	2.4 ± 1.6 cm^2^; 3, 4 stages (Modified Berndt Harty radiologic classification)	39.5 ± 18.4	Group (1): ↓ VAS; ↑ AOFAS. MOCART = 53 ± 16
Usuelli, 2018 [17]	Prospective case series NO	Group (1): COLL I/III + autologous BG	AR	20/20	36.1 ± 13.1	11/9	24.6 ± 2.7	1.5 ± 0.9 cm^2^; 3, 4 stages (Modified Berndt Harty radiologic classification)	24	Group (1): ↑ AOFAS, MOCART; ↓ VAS
Sadlik, 2017 [51]	Prospective case series NO	Group (1): COLL I/III + autologous BG+BMC	MMO	10/10	37 ± 12.5	6/4	26.7 ± 3.5	1.3 ± 0.6 cm^2^; n.r.	46.4 ± 18	Group (1): ↑ AOFAS; ↓ VAS. MOCART = 70 ± 17
López-Alcorocho, 2021 [19]	Prospective case series Single-blind (Clinical assessor)	Group (1): COLL I/III + high-density chondrocytes	AR or MMO	24/24	Mean 31	14/10	n.r.	2.1 ± 0.6 cm^2^; 3, 4 grade (ICRS)	24	Group (1): ↓ VAS; ↑ AOFAS. MOCART = 72 ± 16
Drobnic, 2021 [52]	Prospective case series NO	Group (1): Aragonite-based bi-phasic	AR or MMO	4/6	Mean 42	2/2	33.6 ± 4.4	2.0 ± 0.1 cm^2^; Stage IV, V (Hepple’s classification)	Mean 26	Group (1): ↑ FAOS
Younger, 2016 [53]	Prospective, case series NO	Group (1): β-TCP matrix + rhPDGF	AR	5/6	52 ± 8.5	2/3	26.3 ± 5.0	1.0 ± 0.4 cm^2^; n.r.	6	Group (1): ↓ VAS; ↑ AOS. MOCART = average 68
Sadlik, 2019 [54]	Prospective comparative NO	Group (1): Autologous bone chips covered with COLL I/III or HA-based + BMC + MMO; Group (2): Autologous bone chips covered with COLL I/III or HA-based+BMC+AR	AR or MMO	24/24	Mean 34.1	14/10	Mean 25.2	Group (1): 1.3 ± 0.6 cm^2^ Group (2): 1.2 ± 0.4 cm^2^; n.r.	Mean 22	Groups (1), (2): ↑ AOFAS; ↓ VAS; =MOCART. No differences between groups
Guney, 2015 [55]	RCT Single-blind (radiologist assessors)	Group (1): PRP; Group (2): no treatment	AR	35/43	Mean 40.7	16/19	Mean 27.5	n.r.; n.r.	Mean 16.2	Group (1): ↓ VAS; ↑ AOFAS, FAAM than group (2)

**ABBREVIATIONS:** COLL = Collagen; BG = bone graft; MMO = Medial malleolar osteotomy; Pts = patients; SD = standard deviation; M = male; F = female; BMI = body mass index; OCL = osteochondral lesion; n.r. = not reported; f-up = follow-up; m = months; VAS = Visual Analogue Scale; AOFAS = American Orthopedic Foot and Ankle Society; MOCART = Magnetic Resonance Observation of Cartilage Repair Tissue; M-BMS = matrix-augmented bone marrow stimulation; FAAM-ADL = Foot and Ankle Ability Measure-activity daily living; FAOS = Foot and Ankle outcome score; QoL = Quality of Live; AR = arthroscopy; HA-based = hyaluronic acid-based; Open = open surgery; MRI = Magnetic Resonance Imaging; PGA-HA = polyglycolic acid-hyaluronan; KAFS = Karlsson and Peterson Scoring System for Ankle function; PJCAT = particulated juvenile cartilage allograft transplantation; PRP = Platelet Rich Plasma; CFS = cell-free scaffold; JACI = juvenile allogenic chondrocyte implantation; BMAC = bone marrow aspirate concentrate; FFI = Foot Function Index; BMC = bone marrow concentrate; β-TCP = β-tricalcium phosphate; rhPDGF = recombinant human platelet derived growth factor; RCT = randomized control trial; Yrs = years; ↑ = high; ↓ = low.

**Table 2:** Evaluation of the included studies using the Downs and Black checklist and the modified Coleman Methodology Score. The first 6 columns regard the Downs and Black checklist and the following 3 columns the modified Coleman Methodology Score.

	Downs and Black Checklist						Modified Coleman Methodology Score		
Article	Reporting	External Validity Bias	Internal Validity Bias	Internal Validity Confounding	Power	Total Score	Part A	Part B	Total Score
Weigelt, 2019 [28]	10	2	5	3	0	**20**	39	10	49
Gottschalk, 2021 [29]	9	2	5	3	0	**19**	34	10	44
Ayyaswamy, 2021 [30]	9	2	5	3	1	**20**	24	10	34
Yontar, 2019 [31]	7	2	5	2	0	**16**	21	10	31
Giannini, 2014 [32]	8	2	5	4	0	**19**	42	10	52
Di Cave, 2017 [33]	8	2	5	2	0	**17**	37	10	47
Kanatlı, 2017 [34]	9	2	6	2	1	**20**	42	10	52
Albano, 2017 [35]	9	2	5	2	0	**18**	33	10	43
Li, 2021 [36]	8	2	5	2	0	**17**	37	10	47
Georgiannos, 2016 [37]	8	2	5	2	0	**17**	37	10	47
Sawa, 2018 [38]	10	2	5	3	0	**20**	25	10	35
Heida, 2020 [39]	8	2	5	2	0	**17**	24	10	34
Akmeşe, 2020 [40]	9	2	5	1	0	**17**	50	10	60
Camurcu, 2020 [41]	9	2	6	2	1	**20**	45	10	55
Dogar, 2021 [42]	9	2	5	2	0	**18**	35	10	45
Gorgun, 2022 [43]	9	2	6	3	0	**20**	50	10	60
Kekeç, 2023 [44]	9	2	5	2	0	**18**	42	10	52
Eren, 2019 [45]	8	2	6	2	1	**19**	33	10	43
Karnovsky, 2018 [46]	9	2	5	2	0	**18**	37	10	47
Christensen, 2016 [47]	8	2	6	2	0	**18**	45	10	55
Gottschalk, 2017 [48]	9	2	5	3	0	**19**	39	10	49
Galla, 2019 [49]	10	2	5	2	1	**20**	49	10	59
Kubosch, 2016 [50]	9	2	5	2	0	**18**	45	10	55
Usuelli, 2018 [17]	9	2	5	2	1	**19**	47	10	57
Sadlik, 2017 [51]	8	2	5	2	0	**17**	45	10	55
López-Alcorocho, 2021 [19]	9	2	6	2	0	**19**	56	10	66
Drobnic, 2021 [52]	8	2	5	2	0	**17**	32	10	42
Younger, 2016 [53]	8	2	5	2	0	**17**	38	10	48
Sadlik, 2019 [54]	10	2	5	3	0	**20**	43	10	53
Guney, 2015 [55]	9	3	6	4	1	**23**	41	10	51

**Table 3:** Main complications after surgical and scaffold applications. The studies were divided according to surgical approach type: (1) AR; (2) MMO; (3) Open surgery.

Surgical Technique	Scaffolds	No Scaffold	Article
AR	Hyaluronan + chondrocytes: Re-operation (6.5%)		Giannini, 2014 [32]
	PGA-HA: Ankle swelling (9.4%)		Kanatlı, 2017 [34]
	Osteochondral autograft: NO		Li, 2021 [36]
	PJCAT allograft: Re-operation (6.1%)		Heida, 2020 [39]
	HA-based: Re-operation (2.4%); Chitosan-based: Re-operation (2.6%)		Akmeşe, 2020 [40]
	Chitosan-glycerol phosphate: Hematoma of the ankle (6.3%); erythema and swelling (3.1%)	Hematoma of the ankle (6.5%)	Camurcu, 2020 [41]
	Chitosan-based: NO	Transient neurapraxia in the dorsal branch of the superficial peroneal nerve (4.5%); Mosaicplasty due to persistent pain (9.01%)	Dogar, 2021 [42]
	COLL I/III + autologous BG: Superficial skin infection of the arthroscopic portal (4.3%); Autologous BG: Superficial skin infection of the arthroscopic portal (2.1%); synovial fistula (2.1%)		Gorgun, 2022 [43]
	Polyglycolic acid-hyaluronan: NO	NO	Eren, 2019 [45]
	PJCAT + JACI-BMAC: Re-operation (25%): BMAC: Re-operation (30%)		Karnovsky, 2018 [46]
	COLL I/III + autologous BG: Re-operation (5%)		Usuelli, 2018 [17]
	β-TCP matrix + rhPDGF: NO		Younger, 2016 [53]
MMO	COLL I/III + autologous BG: Delayed union (3%); Re-operation (57.6%)		Weigelt, 2019 [28]
	MaioRegen: Re-operation (25%)		Albano, 2017 [35]
	Autologous BG: Superficial wound infection (6.5%); Numbness at the distribution area of superficial peroneal nerve (2.2%); Occasional ache over the anteromedial aspect of ankle (4.3%)		Georgiannos, 2016 [37]
	Autologous BG: NO		Sawa, 2018 [38]
	COLL I/III + autologous BG: NO		Kubosch, 2016 [50]
	COLL I/III + autologous BG + BMC: Iatrogenic lesion of the posterior tibial tendon (10%)		Sadlik, 2017 [51]
Open surgery	COLL I/III + autologous BG: Transient p.o. irritation of the deep peroneal nerve (4.3%); painful arthrofibrosis (4.3%); persistent pain (4.3%)		Galla, 2019 [49]

**Table 4:** Positive correlations between patients/lesions features and clinical results. NO = studies that analyzed the correlations, but did not find a correlation; / = studies that did not take the correlation into consideration.

Article	Age	BMI	Gender	Duration of Symptoms	P.o. Pain	Lesion Size	Lesion Location
Giannini, 2014 [32]	**<40 yrs and high AOFAS** (*p* = 0.046 at 12 mo; *p* = 0.05 at 36 mo; *p* = 0.008 at final f-up)	/	/	/	/	/	**Lateral lesion and high AOFAS** (*p* = 0.007 at 12 mo, *p* = 0.001 at 36 mo)
Gottschalk, 2017 [48]	/	/	/	/	/	**Lesion size and FFI pain and function** at final f-up (*p* = 0.012, *p* = 0.016)	/
Kubosch, 2016 [50]	**≥45 yrs and low pain** (*p* = 0.048)	**BMI > 30 and low AOFAS Score** (*p* = 0.003); **BMI > 30 and high VAS** (*p* = 0.031)	/	/	**High p.o. pain and low AOFAS** (*p* = 0.004)	**Lesion size ≥ 3 cm^3^ and low AOFAS** (*p* = 0.041)	/
Weigelt, 2019 [28]	NO	NO	/	/	/	NO	/
Gottschalk, 2021 [29]	NO	NO	NO	NO	/	/	NO
Sadlik, 2017 [51]	/	/	/	/	/	NO	/
Ayyaswamy, 2021 [30]	NO	/	/	/	/	NO	NO
Sawa, 2018 [38]	/	/	/	/	/	NO	/

## Data Availability

The original contributions presented in the study are included in the article material, further inquiries can be directed to the corresponding author.
